# External and Total Hip Rotation Ranges of Motion Predispose to Low Back Pain in Elite Spanish Inline Hockey Players

**DOI:** 10.3390/ijerph17134858

**Published:** 2020-07-06

**Authors:** Antonio Cejudo, Víctor Jesús Moreno-Alcaraz, Ricardo Izzo, Fernando Santonja-Medina, Pilar Sainz de Baranda

**Affiliations:** 1 Department of Physical Activity and Sport, Faculty of Sport sciences, Regional Campus of International Excellence “Campus Mare Nostrum”, University of Murcia, 30720 San Javier (Murcia) C.P., Spain; antonio.cejudo@um.es (A.C.); victorjm@um.es (V.J.M.-A.); 2 Sports and Musculoskeletal System Research Group (RAQUIS), University of Murcia, 30720 Murcia, Spain; riccardo.izzo@uniurb.it; 3 Dipartimento di Scienze Biomolecolari, Scuola di Scienze Motorie, Università degli Studi, 61029 Urbino C.P., Italy; 4 Department of Surgery, Pediatrics, Obstetrics and Gynecology, Faculty of Medicine, Regional Campus of International Excellence “Campus Mare Nostrum”, University of Murcia, 30100 Murcia C.P., Spain; 5 Traumatology and Orthopedic Surgery Service, Virgen de la Arrixaca University Clinical Hospital, 30120 El Palmar (Murcia) C.P., Spain

**Keywords:** assessment, ROC curve, ROM-SPORT battery, injury prevention, back pain

## Abstract

Low back pain (LBP) is a common ailment in competitive athletes. Although the association between limited range of motion (ROM) and prevalence of LBP has been widely investigated in other sports, there is no research about this topic in inline hockey (IH). The main purposes of this study in IH players were (1) to analyze the association between hip ROM and LBP and (2) to establish a diagnostic cutoff for ROM of high risk of LBP. Twenty elite IH players from the two Spanish National Teams (male and female) were assessed for passive maximum hip ROMs; the movement evaluated were the hip external [HER] and internal [HIR] rotation, hip flexion with flexed [HF-KF] and extended [HF-KE] knee, hip adduction with flexed hip [HAD-HF], hip abduction with neutral [HAB] and flexed [HAB-HF] hip, and hip extension [HE]. LBP was retrospectively monitored for the last 12 months before the date of ROM assessment by using a questionnaire. The data were analyzed via a binary logistic regression and receiver operating characteristic curves. The 70% of players had developed LBP during the retrospective study period. Significant differences between LBP group and asymptomatic group for HER (*p* = 0.013, d [Cohen’s effect size] = 1.17) and hip total rotation [HTR] (*p* = 0.032, d [Cohen’s effect size] = 1.05) were observed. The cutoff points with the greatest discriminatory capacity were 56.5° for HER and 93° for HTR ROMs.

## 1. Introduction

The popularity of inline hockey (IH) has increased in Spain over the last few years, which is reflected in an increase in the number of federal licenses, national leagues, and sports teams [1]. The increased participation of IH players is likely to lead to an increase in the prevalence of injuries [2]. The injuries reduce athletic performance and produce lost time of training and competition for long periods [3,4,5]. 

Previous studies have correlated inline hockey (IH) with a high risk of sport injury [3,4,5]. Hutchinson et al. [4] and Moreno-Alcaraz, Cejudo, and Sainz de Baranda [3] showed an estimated game injury rate of 139 and 300 injuries per 1000 athlete-exposures, respectively. Varlotta et al. [5] reported a game injury rate of 304.9 injuries per 1000 game hours. Lacerations, contusions, strains, and sprains are the most prevalent injuries in professional IH players [3,4,5]. The most frequently injured body regions are the head/neck, shoulder, knee, ankle, and back [3,4,5]. The most prevalent injuries in professional IH player are knee sprains and lumbosacral sprain [5] and lower trunk contusions [3]. In addition, previous epidemiological data of injuries in IH showed the lumbar spine to be one of the body sites frequently affected, especially via non-contact mechanisms [4,5,6]. Although, the lumbosacral region is one of the most affected regions in IH-related injuries, little is referred in the literature to the incidence of low back pain (LBP).

The LBP is a very common ailment in competitive athletes [7,8,9,10]. Given the biomechanical relationships between the hip, pelvis, and spine, especially the multiple shared muscles (psoas, quadratus lumborum, erector spinae, gluteus maximus, etc.), the hip joint has been considered as a main contributor to LBP [11,12,13]. It has been demonstrated that restricted range of motion [ROM] increases the risk of injury in athletes. One of the most studied is the association between the restricted hip flexion with extended knee (hamstring tightness) and the LBP in tennis players [14], soccer players [15], and runners [16]. The limited hip extension ROM [17,18,19] and hip internal rotation ROM or hip total rotation ROM was associated with LBP in professional tennis players [10,14], professional golfers [9], athletes of a rotation-related sports [20], amateur golfers [21], and judo athletes [22]. Also, several studies have found relation between LBP and limited hip internal and external rotation and hip total rotation in the non-athletic population of different ages and sex [23,24,25,26]. In this sense, asymmetric (side-to-side) hip rotation ROM is also a risk factor for LBP in different sports [9,21,24,27,28,29]. The importance of the association between LBP and hip rotation ROMs is based on the hypothesis that a limited hip ROM results in compensatory motions of the lumbopelvic region. These compensatory motions generate an increase of the loads and stress in the join tissues of the lumbopelvic region and, as a consequence, produce eventual LBP symptoms [9,25,26,30]. All these studies show a significant relationship between limited hip ROM and LBP, but the muscle groups involved in this relationship remain unclear. Clinical and sports professionals hold the supposition that the tightness of muscles inserted in the pelvis (such as the iliopsoas, gluteus maximus, hip adductors, or piriformis) alters the pelvis disposition and the spinal morphotype and, therefore, predisposes to LBP [11]. Although the association between limited ROM and prevalence of LBP has been widely investigated in other sports [20,31,32,33,34,35,36,37], there is no research about this topic in IH. 

Regarding the technical skills of IH, skating is the most important skill and represents a specific pattern of movement of this sport. Skating stride requires good levels of ankle plantarflexion and dorsiflexion, knee flexion and extension, hip extension and flexion, hip adduction and abduction, and hip external and internal rotation [38,39,40]. The repetition of skating movements may cause certain musculoskeletal adaptations in IH players such as muscular imbalances, tightness, and asymmetry, causing abnormal pelvis and spinal sagittal alignment and predisposing to LBP [41,42,43,44,45,46]. 

However, it is unknown which movements are affected by these negative adaptations in IH and which modified movements are in relation with LBP.

Considering all the above mentioned, the starting hypothesis was that IH players with LBP would have less hip ROM and more asymmetry than asymptomatic IH players. Thus, the main purposes of this study in IH players were (1) to analyze the association between hip ROM and LBP and (2) to establish a diagnostic cutoff for ROM of high risk of LBP. 

## 2. Method

This investigation was a retrospective cohort study of LBP in IH players. The association of demographic data, training regimen, and hip ROMs measures with the incidence of LBP in elite Spanish IH players was examined. This study was performed in IH players that displayed LBP during the last 12 months (*n* = 14) and in IH players without history of LBP during this period (*n* = 6). Finally, we established cutoff values for the associated variables that identify high risk of LBP. 

### 2.1. Participants

The participants of these study were IH players of the Spanish National Team who took part in the preparative technical meeting (or training camp) prior to the Roller Games World Championship in Nanjing in 2017. The recruitment lasted the first three days of the technical meeting. Twenty-six elite Spanish IH volunteer players (13 males and 13 females) between the ages of 18 and 29 years (22.50 ± 2.89 y) participated in the study. The sample was homogeneous in potential confounding variables, except in training days (*p* = 0.31) and training months (*p* = 0.007) per year (Table 1). The training volume defined as “Training hours during the last 12 months” was calculated using the formula: Training hours × day × weekly training days × 4 weeks per month × months per year [47,48,49]. None of the participants was involved in a systematic and specific stretching regimen in the previous six months. Besides, the participants did not usually perform stretching exercises daily during their warm-up or cool-down phases.

None of the participants had orthopedic problems affecting the knee, thigh, hip, or lower back in the previous three months the could have impacted the players´ habitual movement competency and/or lower extremity ROM profile. Four goalkeepers (2 males and 2 females) and 2 male junior IH players were excluded. Goalkeepers were excluded because they show significant physiological differences (body composition, anaerobic power, strength, and flexibility) to field (defense and forward) hockey players [51]. Two junior inline hockey were excluded because these players had been selected to assist in training and did not have the same competitive level. Finally, data from 20 players were analyzed (Table 1).

The measures of hip ROM were conducted at the end of the competition phase of the year 2017. Before participation, the experimental procedures and potential risks of the ROM assessment were fully explained to the participants in verbal and written form, and a written informed consent was obtained. The experimental procedures used in this study were in accordance with the Declaration of Helsinki and ethical approval was obtained from the Ethics and Scientific Committee of the University of Murcia (Spain) [ID: 1702/2017].

### 2.2. Procedure

The evaluation session was conducted in the previous technical meeting of the Spanish National Team prior to the participation in the Roller Games World Championship in Nanjing in 2017. All the IH players were asymptomatic in the evaluation session. Both the questionnaire (demographic, sport-related background, training regimen, and previous history of LBP) and hip ROM were conducted the first three days of the technical meeting. Six or seven players were measured each day. Anthropometric traits (weight, height, body mass index, and body fat) and ROMs (hip, knee, and ankle) were assessed by two experienced examiners.

### 2.3. Questionnaire

Before the ROM measurement, the participants completed a questionnaire based on previous studies including information related to sport background (tactical position, current competitive level, dominant lower extremity, sport experience), anthropometric traits (body mass, stature, and body mass index), and training regimen (weekly practice frequency, hours of IH practice per week and day). The LBP-related questions was based on standardized, validated, and internationally accepted Nordic questionnaires to study the prevalence of occupational symptoms [52,53]. The following standard questions from the Nordic questionnaire were included:

- Have you ever experienced LBP?
- Have you ever experienced LBP during the previous seven days?
- How many days during the last 12 months have you had LBP?
- Have you been examined or treated for LBP by a physician, physical therapist, chiropractor, or other health personnel as an outpatient during the previous 12 months?

Low back pain was defined as “pain, ache, or discomfort in the lower back with or without radiation to one or both legs”. LBP was considered when IH players had experienced any LBP limiting their performance for a period greater than three days. LBP lasting for 48–72 hours was considered as “delayed onset muscle soreness” [9].

### 2.4. ROM-Sport Battery

**Figure 1:**
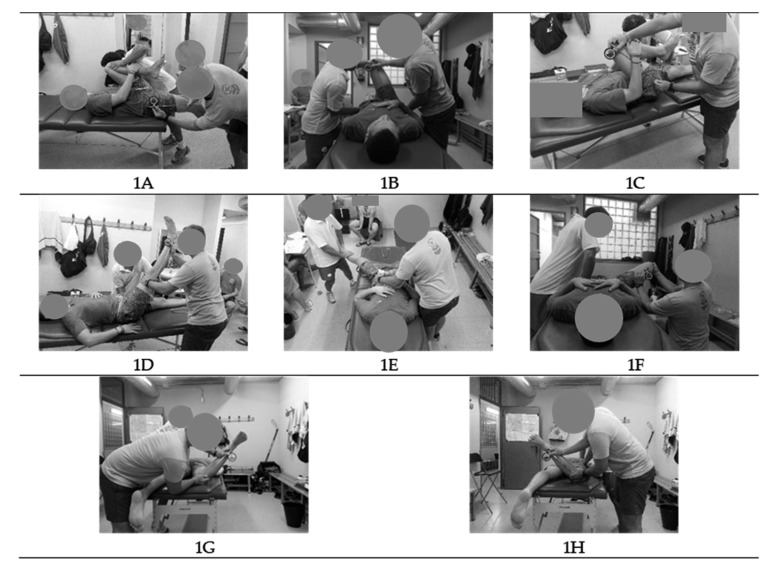
Lower extremity hip ranges of motion of the ROM-Sport Battery: Hip extension test [HE] (**A**), hip adduction with hip flexed 90° test [HAD-HF] (**B**), hip flexion with knee flexed test [HF-KF] (**C**), hip flexion with knee extended test [HF-KE] (**D**), hip abduction with hip neutral test [HAB] (**E**), hip abduction with hip flexed 90° test [HAB-HF] (**F**), hip internal rotation test [HIR] (**G**), hip external rotation test [HER] (**H**); ROM: range of motion.

Measures of eight passive hip ROMs were taken. The maximum passive hip extension [HE] (iliopsoas, Figure 1A), hip adduction with hip flexed 90° [HAD-HF] (piriformis, Figure 1B), hip flexion with knee flexed [HF-KF] (gluteus maximus, Figure 1C) and extended [HF-KE] (hamstring, Figure 1D), hip abduction with hip neutral [HAB] (adductors, Figure 1E) and hip flexed 90° [HAB-HF] (monoarticular adductors, 1F), hip internal rotation [HIR] (external rotators, 1G), hip external rotation [HER] (internal rotators, 1H), and hip total rotation [HTR] ROMs of the dominant and non-dominant leg were assessed following the methodology previously described (ROM-Sport Battery) [54] (Figure 1). The value of HTR was obtained from the sum of the value of HIR and the value of HER.

These tests were selected based on previous reliability [55,56] and validity [57,58] studies, and also based on the anatomical knowledge and extensive clinical and sport experience [59,60]. Furthermore, these tests have been considered appropriate by the American medical organizations [59,61] and included in manuals of Sports Medicine and Science [59,60,62,63,64]. In addition, studies from our laboratory have reported moderate to high reliability (variability ranging from 4° to 7°) for the procedures employed [65,66].

One familiarization session and one testing session were made. The familiarization session was performed one week before the study. The purpose of the familiarization session was explaining to the players the correct technical execution of the exploratory tests. The dominant leg was defined as the participant´s preferred kicking leg [54]. All tests were carried out by the same two experienced examiners (one conducted the tests and the other ensured proper testing position of the participants) under stable environmental conditions. The sport scientists were blinded to the purpose of the study.

Prior to the testing session, all participants performed the dynamic warm-up designed by Taylor et al. [67]. The warm-up lasted approximately 15–20 min. A 3–5-min rest interval between the end of the warm-up and beginning of the ROM assessment was given to the IH players; this was the time needed to get hydration and to dry their sweat before ROMs’ assessment [68]. It is important to note that it has been shown that the effects elicited by the dynamic warm-up on the muscle properties lasted more than 5 min [69] and, hence, decreases in ROM values within the 3–5-min rest interval were not expected.

After the warm-up, IH players were asked to perform two repetitions of each ROM test for each leg. The mean score for each test was used in the analyses. The ROM tests were performed in a randomized order. The IH players were examined wearing sports clothes and without shoes. They were allowed to rest for 30 s between repetitions, legs, and tests.

Lower extremity hip ranges of motion were measured using an ISOMED Unilevel inclinometer (Portland, Oregon) with an extendable telescopic rod [65]. Before each assessment session, the inclinometer was calibrated to 0° either with the vertical or horizontal. The angle between the longitudinal axis of the mobilized segment was recorded (following its bisector) with the vertical or the horizontal [65,69]. A metal goniometer with a long arm (Baseline® Stainless) was used to measure the hip abduction movement with a hip neutral and a lumbar support (Lumbosant, Murcia, Spain) to standardize the lumbar curvature [70,71]. 

The endpoint for each test was determined using at least one of the following criteria: (1) An examiner felt or appreciated some compensation movement that increased the ROM onset of pelvic rotation and/or (2) the IH player felt a strong but tolerable stretch, slightly before the occurrence of pain [65].

### 2.5. Statistical Analysis

Before the statistical analysis, the distribution of raw data sets was checked using the Shapiro-Wilk tests to determine normality of data. The Levene’s test was used to assess homogeneity of variance between groups (male vs. female). The results demonstrated that all data had an abnormal distribution. 

Descriptive statistics including means and standard deviations were calculated for all variables studied. Data were analyzed using independent sample of the Mann–Whitney U test to examine possible differences in demographic variables and hip ROMs between the male and female groups. In addition, the Wilcoxon test was carried out to assess differences between the values of the dominant and non-dominant limbs (asymmetry). Additionally, Cohen’s effect size was calculated for all ROM results. The magnitude of the effect size was classified as previously described by Hopkins et al. [50] as trivial (<0.2), small (0.2 to 0.59), moderate (0.6 to 1.19), large (1.20 to 2.00), very large (2.00 to 3.99), or extremely large (>4.0). Asymmetry was considered when the magnitude of the effect size was moderate, which is established as the minimum level of relevant effect with practical application [50] or higher than moderate. 

The Mann–Whitney U test was performed to compare the continuous variables (anthropometric characteristics, sport-related background, and training regimen variables, eight hip ROMs, and the eight hip ROMs’ asymmetry) between the IH players displaying LBP and those asymptomatic. Additionally, Cohen’s effect size was calculated for all ROM results, and the magnitudes of the effect were classified as described above. 

The relationship between the independent variables and the dependent variable was examined by backward stepwise binary logistic regression (forward selection [conditional], inclusion probability *p* ≤ 0.05, elimination probability *p* ≤ 0.10) with OR (Odds ratio) analysis was used as in previous studies [33,72,73] for estimating the simultaneous effects of several predictors instead of relative risk estimates [74]. Effect sizes for the OR were defined as follows: Small effect OR = 1–1.25, medium effect OR = 1.25–2, and large effect OR ≥ 2 [75].

To determine whether it was possible to find a clinically relevant cutoff point for ROM that could be used for pointing out individuals at high risk for LBP, receiver operating characteristic (ROC) curves were calculated. The area under the ROC curve represented the probability that a selection based on the risk factor for a randomly chosen positive case would exceed the result for a randomly chosen negative case. The area under the curve could range from 0.5 (no accuracy) to 1.0 (perfect accuracy). If it was found to be statistically significant, it meant that using the risk factor as a determinant was better than guessing. Since the ROC curve plots sensitivity against 1 minus specificity, the coordinates of the curve can be considered possible cutoff points, and the most suitable cutoff can be chosen.

Among the IH players who sustained LBP, Pearson’s chi-squared test was used to examine the existence of a relationship between the ROM classification (normal and limited) and LBP. Analysis was performed using the SPSS version 20 software (SPSS Inc, Chicago, IL, USA). For all analyses, significance was accepted at *p* < 0.05. Data were presented as means ± standard deviation.

## 3. Results

Significant differences in ROM according to sex (Table 2) were observed in HAD-HF (*p* [statistical difference value] = 0.019, d [Effect sizes Cohen’s d] = −1.2297), HER (*p* = 0.029, d = −1.2297), HAB-HF (*p* = 0.005, d = −1.4821) and HF-KF (*p* = 0.002, d = −1.823). ROM asymmetry of the hip was observed in HF-KF (*p* = 0.042, d = 0.724) in male IH players and HAB (*p* = 0.005, d = 1.176) and HTR (*p* = 0.016, d = 0.680) in female IH players, with the lower values in the non-dominant limb (Table 2). 

Of the 20 IH players included in this study, 14 IH players experienced LBP at least once during the previous 12 months. Players with history of LBP and asymptomatic ones had similar training volume during the study period. There were statistically significant differences and effect size (d > 0.6) between LBP group and asymptomatic group in HER [LBP group 62.1 ± 8.2° vs. asymptomatic group 53.2 ± 5.5°, *p* = 0.013, d = 1.17 (large effect sizes)] and HTR [LBP group 101.3 ± 12° vs. asymptomatic group 89.5 ± 8.7°, *p* = 0.032, d = 1.05 (moderate effect sizes)]. The group of IH players with LBP had an increased range of 8.9° and 11.8° in the HER and HTR, respectively (Table 3).

Stepwise logistic regression analysis showed that of all variables described in Table 3 entered into the model (Table 4), only HER showed small predictors of LBP occurrence in the 20 IH players (OR = 1.174 [small], 95% CI [Confidence Interval]= 0.995 to 1.386, *p* = 0.057). In addition, the analysis of the frequencies showed 85.7% of successful cases in 20 IH players with LBP who were categorized with high HER (high extensibility of internal rotator muscles, cutoff ≥ 56.5°), according to the present study. None of the other intrinsic factors imposed a significant relative risk for LBP (*p* > 0.05).

**Figure 2:**
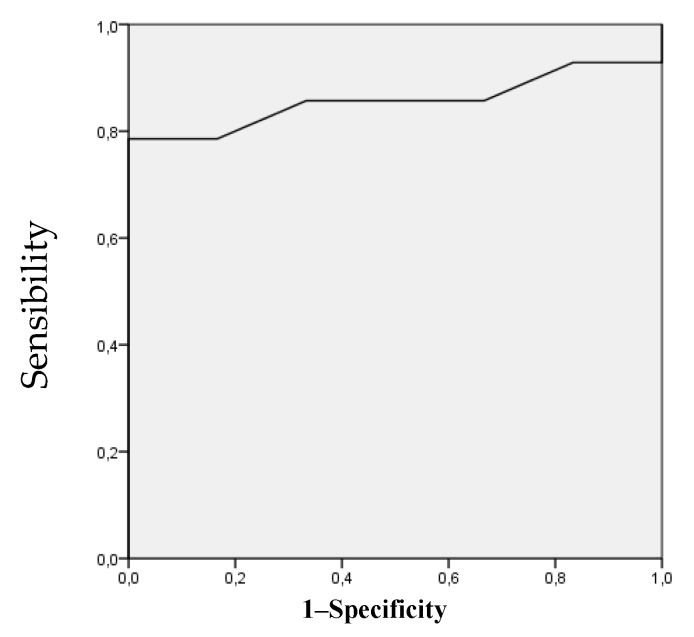
Receiver operating characteristic curve analysis for the hip external rotation ROM as a risk factor for lower back pain. The area under the curve is 0.857 (*p* = 0.013); the coordinates represent possible cutoff point in hip external rotation range of movement (the optimal cutoff point was 56.5°).

**Figure 3:**
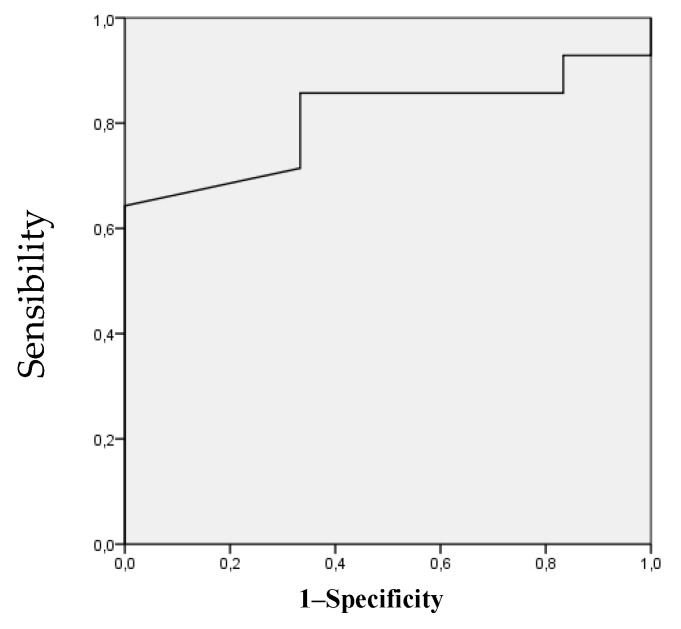
Receiver operating characteristic curve analysis for the hip total rotation ROM as a risk factor for lower back pain. The area under the curve is 0.810 (*p* = 0.032); the coordinates represent possible cutoff point in hip total rotation range of movement (the optimal cutoff point was 93°).

Two movements showed a good accuracy of the predictive model for LBP [76]. The area under the ROC curves for HER and HTR was 0.857 and 0.810, respectively (Figure 2 and Figure 3), being statistically significant (HER [*p* = 0.013, standard error: 0.087, 95% confidence interval: 0.687 to 1.000], HTR [*p* = 0.032, standard error: 0.097, 95% confidence interval: 0.619 to 1.000]). Using the coordinates of the curves, the angles of HER and HTR that most accurately identified individuals at risk for LBP were 56.5° (sensibility 0.857 and 0.333 specificity) and 93° (sensibility 0.857 and 0.333 specificity), respectively.

Finally, the Pearson’s chi-squared test showed significant differences between the proportions of IH players with high and normal ROM for HER and HTR, and players with LBP and asymptomatic (*p* = 0.019, 95% confidence interval 1.248 to 115.362). IH players with HER ≥ 56.5° and HTR ≥ 93° have 12 times more risk of developing LBP than IH players with HER < 56.5° and HTR < 93°.

## 4. Discussion

### 4.1. Tightness as an Intrinsic Risk Factor for the Development of Low Back Pain [LBP]

The review of the literature shows that age, body mass index, sex, body mass, height, years of experience as an IH player, training hours per week, competitive level, long sticks, ROM, or asymmetry are suggested as possible risk factors for LBP [21,43,45,77,78,79,80]. In the current study, we found significant differences between both groups of players (LBP vs. asymptomatic) with respect to the HER (*p* = 0.013, d = 1.17 [large effect sizes]) and HTR (*p* = 0.032, d = 1.05 [large effect sizes]) in the descriptive analysis. Interestingly, the IH players with LBP had higher values in both ROMs than the asymptomatic ones. In addition, this result was confirmed by the regression model, which showed that the only predisposing factor for the development of LBP in IH players was a HER higher than 56.5°. This unexpected result is the main finding of our study.

Interestingly, these results differed from those reported previously, where the association between the limited hip ROM and LBP has been investigated in several sports. Van Dillen, Bloom, Gombatto, and Susco [20] showed that racket sports and golf athletes with a chronic or recurrent LBP history displayed significantly less HIR (LBP 27.7° vs. asymptomatic 30.4°) and HER (LBP 26.5° vs. asymptomatic 30.8°) than those without LBP history. In this sense, two studies including professional golfers and tennis players conducted by Vad et al. [9,10] also found a deficit in HIR in those athletes displaying LBP compared with asymptomatic players (11.8° vs. 16.9°, 16.1° vs. 24.2°). This observation has been also made in amateur golf athletes (21.1° vs. 31.1°) in active and passive hip rotation tests [21]. Similar results have been reported in judo [22], showing the athletes with LBP have limited active HIR (39.5° vs. 46.7°) on the dominant side and HTR (97.6° vs. 105.1°) compared to those without LBP and reduced passive HIR (dominant limb, 41.9° vs. 46.1°, non-dominant limb, 37.1° vs. 47.3°) in both sides and HTR (98.5° vs. 105°). The results observed in athletes with LBP in these studies may be explained by the high training loads and repeated technical movements used in training and competition during the sports’ season, when the recovery measures and recovery time are not sufficient [43,81,82]. This situation produces musculoskeletal adaptations such as muscle tightness, limited ROM, and muscle imbalance that predispose to LBP [43,83,84]. 

The differences between our study and those reported previously may be explained by the sport-specific movements performed by athletes. In this sense, a large number of studies have confirmed that flexibility is specific to each sport [85,86] and tactical position [87,88] and may also depend on the competitive level of the players [89,90]. Skating is a unique pattern of movement in sport and the most important basic skill in IH. Inline skating is characterized by the continuous movement of hip internal and external rotation with the players turning, stopping, crossing over, skating from start propulsion, or transitioning from start to maximum speed [39]. During the sliding phase, when the skate is set on the ground and players are progressively loading all their weight onto their pushing leg, progressive flexion of the hip and knee as well as ankle dorsi-flexion are performed. The push-off phase starts in the middle of the sliding phase, while leaning on the pushing leg, and involves hip external rotation, abduction, and extension, knee extension, and ankle plantar flexion. The later recovery phase of the pushing leg involves hip adduction, flexion, and internal rotation, knee flexion, and ankle dorsiflexion [38,39,91]. These technical features of skating result in very wide hip ROMs in order to achieved maximum performance. The increment of hip flexibility, especially in the iliopsoas, adductors, hamstring, gluteus maximus, or piriformis, improve speed, power, and efficiency of skating [39,92,93]. As a consequence, the hip rotation ROMs in IH players (HER 53.2° and HIR 36.3°) are much higher than those reported in tennis [10,11,12,13,14,15,16,17,18,19,20,21,22,23,24,25,26,27,28], racket sports [20], judo [22], and golf athletes [9,20]. 

However, extremely wide HER and HIR, as observed in our study, may cause compensatory movements in the pelvis and the lumbar region [94]. Those players showing LBP in this study probably had altered coordination of hip and lumbopelvic region that resulted in an increase in lumbopelvic-region movements and produced LBP symptoms. It has been already described that lumbopelvic rotation, pelvis anteversion, and the increased lumbar curvature generate lumbar overload and stress and, consequently, LBP symptoms [30,94]. Another important factor affecting the incidence of LBP is the relation between HER and HIR. Usually, the difference between HER and HIR ranges from 5° to 10°, HER being higher than HIR [59,61,63,64]. However, when the difference is higher than 10° (hip pattern III [30]), as occurred in the present study with an average difference between HER and HIR of 23° in LBP players and 16.9° in the asymptomatic ones, there is a higher predisposition to low back pain [30,31,32,33,34,35,36,37,38,39,40,41,42,43,44,45,46,47,48,49,50,51,52,53,54,55,56,57,58,59,60,61,62,63,64,65,66,67,68,69,70,71,72,73,74,75,76,77,78,79,80,81,82,83,84,85,86,87,88,89,90,91,92,93,94,95]. Cibulka et al. [24,95] identified a specific pattern of passive hip rotation motion in people with LBP, which had significantly greater HER than HIR bilaterally, whereas those with evidence of sacroiliac joint dysfunction had significantly greater HER than HIR unilaterally. Therefore, all these facts, which related to the skating technique and the physical demands of IH players, can explain why the high values of HER and HTR found in the present study are related to the incidence of LBP.

### 4.2. Asymmetry as an Intrinsic Risk Factors for the Development of Low Back Pain [LBP]

The ROM asymmetry is also considered an important risk factor for sport injury. In the current study, hip ROM asymmetry was observed in HF-KF, HER, HTR, and HAB, with the lower values in the non-dominant limb. However, the differences in asymmetry magnitude were not significant between the LBP and the asymptomatic group. The results of LBP predictive model revealed that asymmetry was not a predicting factor for LBP in IH players. This sport shows a highly symmetrical performance during skating [96]. In the small proportion of IH players showing asymmetry, it is possible that technical demands result in a higher overload of the non-dominant lower limb, producing a decreased ROM in some movements. Our results related to asymmetry cannot be compared with previous reports about hockey [4,5,43,45,46,51], because asymmetry was not analyzed in those studies.

On the contrary, in other sport disciplines asymmetry has been related to LBP performing descriptive analysis between the right and left extremity. Thus, Sadeghisani et al. [78] described that asymmetry in the HIR and HTR were common findings in patients and athletes with LBP. Van Dillen, Bloom, Gombatto, and Susco [20] reported that athletes of a rotation-related sport with a history of chronic or recurrent LBP displayed more asymmetry in hip rotation (HER and HIR) compared to those without LBP symptoms. In professional golfers [9] and tennis [10] players, Vad et al. found greater asymmetry of HIR in players with LBP. Almeida, de Souza, Sano, Saccol, and Cohen [22] observed that judo athletes with LBP demonstrated hip rotation asymmetry between the dominant and non-dominant side. The different results of these studies and ours could be due to the definition of asymmetry and the statistical analysis used. Also, the dominant and non-dominant extremities should be considered in the analysis due to the flexibility specificity in the sport. 

### 4.3. Practical Considerations

These findings suggest that great attention should be given to the regular assessment of the flexibility during screening of athletes to individually identify players and high risk of LBP by high HER values. Athletes with greater HER often have weakness of the hip internal rotator muscles (tensor fasciae latae and gluteus medius and minimus) [63,64,97]. Hip muscle strengthening exercises are a common intervention to prevent LBP [98]. Stretching and joint mobilization techniques are also commonly used to restore optimal hip ROM in cases of muscle and capsular tightness. In this sense, the IH players with HER and HTR ROM higher than 56.5° and 93° should follow a mixed exercise program (back stretching, hip stretching, core/gluteus strength, and muscle relaxation exercises) to reduce the risk of recurrent LBP in the future [44,99]. One specific aim of this strength/flexibility program should be the balance side-to-side and internal-to-external rotators. Another objective should be the training of the movement quality of the technical skills, especially the coordination of the hip and lumbopelvic region in order to avoid repetition of compensatory movements and the subsequent LBP. A future prospective study with a larger sample would allow studying more variables (i.e., hip, strength, muscle imbalance, dynamic postural control, core stability, training load, etc.) as potential injury risk factors. In addition, the use of machine learning techniques may help to find the best model for predicting LBP with a greater number of risk factors for LBP in IH players.

## 5. Conclusions

The present study demonstrated that HER and HTR are associated with LBP in elite inline hockey players. The cutoff points with the greatest discriminatory power for prognostic screening were those obtained from HER (56.5°) and HTR (93°).

Inline hockey players display higher ROM values than other rotation-related sports such as judo, golf, or tennis. A hip pattern III predominates in IH players, being the difference between HER and HIR higher than reported for the general population and athletes from other disciplines. 

## Figures and Tables

**Table 1:** Demographic and training regimen data of the Spanish National Teams’ inline hockey players.

Variable	Male (*n* = 10)	Female (*n* = 10)	*p*-Value	Effect sizes Cohen’s d ^a^	Total (*n* = 20)
Age (years)	22.30 ± 2.54	22.70 ± 3.33	0.684	−0.1351 (trivial)	22.50 ± 2.89
Body mass (kg)	71.87 ± 9.64	66.83 ± 9.20	0.247	0.5349 (small)	69.35 ± 9.53
Body Height (cm)	1.73 ± 0.05	1.66 ± 0.05	0.005	1.4000 (large)	1.69 ± 0.06
Body mass index (kg/m^2^)	23.96 ± 2.77	24.32 ± 2.88	0.971	−0.1274 (trivial)	24.14 ± 2.76
Years of experience	14.20 ± 2.97	12.90 ± 3.98	0.165	0.3702 (small)	13.55 ± 3.49
Training months/year	11.20 ± 0.92	10.00 ± 0.00	0.007	1.8446 (large)	10.60 ± 0.88
Training days/week	2.80 ± 0.42	2.80 ± 1.03	0.631	0.0000 (trivial)	2.80 ± 0.77
Training hours/week	5.40 ± 2.27	5.00 ± 1.83	0.971	0.1940 (trivial)	5.20 ± 2.02

Data are expressed as mean ± standard deviation; ^a^ Effect size was classified as trivial or no effect (SMD < 0.2), small (SMD = 0.2 to 0.59), moderate (SMD = 0.6 to 1.19), large (SMD = 1.20 to 2.00), very large (SMD = 2.00 to 3.99), and extremely large (SMD > 4.00) according to Hopkins et al. [50]; SMD: standardized mean difference.

**Table 2:** Differences in range of movement according to the sex and asymmetry in Spanish elite inline hockey players (*n* = 20).

Variables	Male (*n* = 10)				Female (*n* = 10)				Male Versus Female	
	Dom	NDom	*p*-Value	ES (d)	Dom	NDom	*p*-Value	ES (d)	*p*-Value	ES (d)
HE (iliopsoas)	7.0 ± 5.8	9.80 ± 7.8	0.132	−0.328 Small	8.2 ± 5.9	10.4 ± 4.40	0.178	−0.441 Small	0.494	−0.1632 Trivial
HAD−HF (piriformis)	25.0 ± 3.3	27.60 ± 5.8	0.128	−0.485 Small	31.2 ± 5.6	32.4 ± 4.7	0.279	−0.220 Small	0.019	−1.2297 Large
HAB (adductors)	39.2 ± 3.2	38.60 ± 3.5	0.560	0.333 Small	41.2 ± 2.9	38.8 ± 3.2	0.005	1.176 Moderate	0.393	−0.3791 Small
HIR (external rotators)	35.0 ± 6.6	37.20 ± 4.6	0.146	−0.392 Small	41.8 ± 6.3	39.2 ± 4.8	0.090	0.392 Small	0.190	−0.8381 Moderate
HER (internal rotators)	57.6 ± 10.9	53.20 ± 8.1	0.068	0.441 Small	64.8 ± 5.3	62.20 ± 6.9	0.057	0.362 Small	0.029	−1.0645 Moderate
HTR (external and internal rotators)	92.6 ± 15.4	90.4 ± 11.7	0.360	0.15 Trivial	106.6 ± 6.0	101.4 ± 8.9	0.016	Moderate 0.680	0.052	−1.1757 Moderate
HAB-HF (adductors monoarticular)	64.0 ± 7.2	65.60 ± 6.1	0.387	−0.153 Small	73.4 ± 5.8	72.60 ± 4.8	0.555	0.220 Small	0.005	−1.4821 Large
HF-KE (hamstring)	71.4 ± 4.2	70.40 ± 4.1	0.322	0.250 Small	74.00 ± 6.6	74.40 ± 6.9	0.716	0 Trivial	0.315	−0.6088 Moderate
HF-KF (gluteus maximus)	133.2 ± 6.3	129.80 ± 5.5	0.042	0.724 Moderate	141.8 ± 6.2	140.20 ± 4.9	0.366	0.196 Trivial	0.002	−1.8239 Large

Dom: Dominant side; NDom: Non-dominant side; ES (d): Effect sizes Cohen’s d; Hip extension test [HE]; hip adduction with hip flexed 90° test [HAD-HF]; hip abduction with hip neutral test [HAB]; hip internal rotation test [HIR]; hip external rotation test [HER]; hip abduction with hip flexed 90° test [HAB-HF]; hip flexion with knee extended test [HF-KE]; hip flexion with knee flexed test [HF-KF]. Effect sizes were was interpreted as trivial or no effect (SMD [standardized mean difference] < 0.2), small (SMD = 0.2 to 0.59), moderate (SMD = 0.6 to 1.19), large (SMD=1.20 to 2.00), very large (SMD = 2.00 to 3.99), and extremely large (SMD > 4.00) [50].

**Table 3:** Comparative analysis of studied variables between inline hockey players displaying LBP and asymptomatic players.

Variables	LBP (*n* = 14)	Asymptomatic (*n* = 6)	*p*-Value	Effect Sizes Cohen’s d
Age (years)	22.71 ± 3.02	22.0 ± 2.76	0.402	Trivial (d = 0.24)
Body mass (kg)	67.11 ± 8.87	74.57 ± 9.69	0.091	Moderate (d = −0.81)
Height (cm)	1.68 ± 0.06	1.73 ± 0.05	0.091	Moderate (d = −0.87)
BMI (kg/m^2^)	23.83 ± 2.83	24.87 ± 2.68	0.444	Small (d = −0.37)
Years of experience	13.71 ± 3.87	13.17 ± 2.64	0.718	Trivial (d = 0.15)
Months of training per year	10.36 ± 0.74	11.17 ± 0.98	0.109	Moderate (d = −0.99)
Days of training per week	2.86 ± 0.86	2.67 ± 0.52	0.841	Small (d = 0.24)
Training hours per week	5.29 ± 2.13	5.00 ± 1.90	0.779	Trivial (d = 0.14)
Training volume (training hours last 12 month)	201.5 ± 83.3	176.0 ± 62.2	0.693	Small (d = 0.32)
HE (iliopsoas)	8.1 ± 4.5°	10.7 ± 7.3°	0.431	Small (d = −0.47)
HAD-HF (piriformis)	30.1 ± 5.4°	26.5 ± 4.1°	0.136	Moderate (d = 0.70)
HAB (adductors)	39.9 ± 3.0°	38.5 ± 2.5°	0.406	Small (d = 0.48)
HIR (external rotators)	39.1 ± 5.7°	36.3 ± 5.2°	0.341	Small (d = 0.5)
HER (internal rotators)	62.1 ± 8.2°	53.2 ± 5.5°	0.013 *	Large (d = 1.17)
HTR (external and internal rotators)	101.3 ± 12.0°	89.5 ± 8.7°	0.032 *	Moderate (d = 1.05)
HAB-HF (monoarticular adductors)	70.4 ± 6.4°	65.5 ± 7.1°	0.186	Moderate (d = 0.74)
HF-KE (hamstring)	72.1 ± 5.4°	73.7 ± 6.2°	0.534	Small (d = −0.28)
HF-KF (gluteus maximus)	137.3 ± 7.2°	133.8 ± 6.6°	0.301	Small (d = 0.49)

Data are expressed as mean ± standard deviation; LBP: Lower back pain; Hip extension test [HE]; hip adduction with hip flexed 90° test [HAD-HF]; hip abduction with hip neutral test [HAB]; hip internal rotation test [HIR]; hip external rotation test [HER]; hip abduction with hip flexed 90° test [HAB-HF]; hip flexion with knee extended test [HF-KE]; hip flexion with knee flexed test [HF-KF]; * significant at *p* ≤ 0.05 (nonparametric Mann–Whitney U test). Effect sizes were was interpreted as trivial or no effect (SMD [standardized mean difference] < 0.2), small (SMD = 0.2 to 0.59), moderate (SMD = 0.6 to 1.19), large (SMD = 1.20 to 2.00), very large (SMD = 2.00 to 3.99), and extremely large (SMD > 4.00) [50].

**Table 4:** Frequencies and logistic regression results: Intrinsic risk factors for lower back pain in the elite inline hockey players.

Variable	Frequencies (%)			Statistics			
LBP (14 vs. 6)		≥56.5°	<56.5°	OR	SE	95% CI	*p*-Value
HER	Asymptomatic	33.3	66.7	1.174	0.085	0.995 to 1.386	0.057
	LBP	85.7	14.3				

HIR: hip internal rotation test; tightness (high risk) establish at 56.5° in the present study; LBP: Lower back pain; OR: Odds ratio (relative risk); OR > 1: Increased odds of shoulder pain; small effect OR = 1–1.25, medium effect OR = 1.25–2 and large effect OR ≥ 2 [75]; SE: Standard error; CI: Confidence interval.
